# Occurrence of maxillary sinus abnormalities detected by cone beam CT in asymptomatic patients

**DOI:** 10.1186/1472-6831-12-30

**Published:** 2012-08-10

**Authors:** Inara Carneiro Costa Rege, Thiago Oliveira Sousa, Cláudio Rodrigues Leles, Elismauro Francisco Mendonça

**Affiliations:** 1 Department of Oral Medicine, Dental School, Paulista University, Goiânia, Goiás, Brazil; 2 Department of Stomatology (Oral Pathology), Dental School, Federal University of Goiás, Goiânia, Brazil; 3 School of Dentistry, Federal University of Goiás, Goiânia, Brazil; 4 Praça Universitária S/N. Setor Universitário, CEP 74605-220, Goiânia, Goiás, Brazil

**Keywords:** Maxillary sinus, Cone beam computed tomography, Abnormality

## Abstract

**Background:**

Although cone beam computed tomography (CBCT) images of the maxillofacial region allow the inspection of the entire volume of the maxillary sinus (MS), identifying anatomic variations and abnormalities in the image volume, this is frequently neglected by oral radiologists when interpreting images of areas at a distance from the dentoalveolar region, such as the full anatomical aspect of the MS. The aim of this study was to investigate maxillary sinus abnormalities in asymptomatic patients by using CBCT.

**Methods:**

1113 CBCT were evaluated by two examiners and identification of abnormalities, the presence of periapical lesions and proximity to the lower sinus wall were recorded. Data were analyzed using descriptive statistics, chi-square tests and Kappa statistics.

**Results:**

Abnormalities were diagnosed in 68.2% of cases (kappa = 0.83). There was a significant difference between genders (p < 0.001) and there was no difference in age groups. Mucosal thickening was the most prevalent abnormality (66%), followed by retention cysts (10.1%) and opacification (7.8%). No association was observed between the proximity of periapical lesions and the presence and type of inflammatory abnormalities (p = 0.124).

**Conclusions:**

Abnormalities in maxillary sinus emphasizes how important it is for the dentomaxillofacial radiologist to undertake an interpretation of the whole volume of CBCT images.

## Background

The multiplanar images acquired by cone beam computed tomography (CBCT) provide an opportunity for radiologists to inspect the entire volume of the acquired image and the anatomic variations and abnormalities that can be found in the image volume [1]. However, this responsibility is frequently neglected when interpreting images of areas at a distance from the dentoalveolar region, such as the full anatomical aspect of the maxillary sinus (MS) [1,2].

Incidental abnormalities of the maxillary sinus are common findings in spiral computed tomography (CT) scans [3-6] and CBCT dental scans [7,8]. In a study by Cha et al., using CBCT examinations, the abnormalities found were signs of acute sinusitis (7.5%), retention cysts (3.5%), and polypoid mucosal thickening (2.3%) [7]. In two other studies, the prevalence of flat mucosal thickening ranged from 23.7% to 38.1%, polypoid mucosal thickening ranged from 6.5% to19.4%, signs of acute sinusitis was 3.6%, and partial and total opacification were 12% and 7%, respectively [8,9].

Failure to detect incidental abnormalities is associated with the limited ability and experience of oral radiologists when interpreting volumetric images and negligence when undertaking a systematic visual scrutiny of the whole image, including the dentoalveolar region and all adjacent structures of the maxillomandibular complex [1,2]. Volumetric images of the maxilla allow for visualization of the entire acquired image volume [1] and the intimate relationship between the upper posterior teeth and the maxillary sinuses as well as the occurrence of certain sinus changes, which are sometimes related to odontogenic causes [10].

Although previous studies reported the occurrence of incidental abnormalities in CBCT scans in patients referred to as orthodontic and other dental purposes [7,8], the prevalence of abnormalities is not known in large samples of scans of patients who underwent the exam for different oral and dental diagnostic purposes. The aim of this study was to investigate the occurrence of maxillary sinus abnormalities in CBCT exams, identify the frequency, type and location of these findings, and the correlation between the distance of periapical lesions and inflammatory changes in the maxillary sinus.

## Methods

The study sample comprises 1113 consecutive records of CBCT tests undertaken at a private radiological clinic in Goiânia, Goiás, Brazil, between November 2006 and December 2008. The research protocol was previously approved by the Local Ethical Committee of the Federal University of Goias (#160/2010).

All the patients had been referred for CBCT diagnosis and treatment planning, which included dental implants, maxillofacial surgery, orthodontics, endodontics, oral pathology, etc. No patient had been primarily referred for a CT scan of the maxillary sinus area because of sinus symptoms or suspected diseases. Gender, age and indication for the exam were recorded.

All CBCT exams which showed the entire maxillary sinuses bilaterally or at least the four sinus walls were included in the study sample, independently of whether the whole maxilla and other anatomical structures were visualized or not. Patients under 12 years old were excluded because of their incomplete sinus development. Images of low resolution quality and/or those in which the presence of metallic artifacts impaired sinus visualization were also excluded.

All CBCT images were taken using the I-CAT Cone Beam 3D imaging system (Imaging Sciences International, Hatfield, PA, USA) using small FOV (6 cm, 8 cm ou 13 cm). Image volume was reconstructed with isotropic-isometric 0.25 × 0.25 × 0.25 mm voxels. The tube voltage was 120 KVp, tube current was 3.8 mA, and an exposure time of 40 seconds was used.

Images were stored and converted into DICOM file format using the acquisition software integrated to the CBCT machine (Xoran, version3.1.62; Xoran Technologies, Ann Arbor, MI, USA). The imported DICOM files were opened and examined using the ImageJ software (ImageJ 1.37v, National Institute of Health, Bethesda, MD, USA). Diagnosis of the maxillary sinuses was performed on a 1:1 scale, using three orthogonal slice views (axial, coronal and sagittal). The PC workstation used the Windows® 7 Home Premium 64-bit (Microsoft Corporation, Redmond, WA, USA), and LG E1950T LED LCD Monitor 18,5" screen size 1360 × 768 pixels (LG Electronics, Seoul, South Korea). ImageJ measuring tools were used to measure mucosal thickening.

The CBCT scans were analyzed by two independent, experienced oral radiologists. The examiners were trained and calibrated using 10% of the sample in a pilot study before data collection began.

A screening procedure was undertaken to identify the presence or absence of sinus abnormalities using a yes/no scale, and orthogonal views of coronal, axial and sagittal scans. The criteria to classify the presence of an abnormality included the identification of at least one of the following deviations from normality: (1) increased or decreased dimension of the sinus, (2) radiographic density changes in the cortical bone of the sinus, (3) partial or complete opacification of the sinus cavity, and (4) increased thickening of the mucosa greater than 3 mm. The screening procedure classified cases as having a sinusal abnormality when both examiners identified at least one type of abnormality, and excluded cases in which both examiners diagnosed the absence of an abnormality. In order to minimize the occurrence of false negative cases in the screening stage, cases were not excluded from the sample when disagreements occurred between the examiners. The identification of an abnormality was registered separately for the right and left sinuses of each patient.

In the next step, selected cases were independently re-evaluated by the two examiners in order to diagnose and classify the cases into different abnormality subtypes, such as congenital changes (aplasia and hypoplasia), malignant and benign tumours, odontogenic lesions (benign odontogenic tumours and inflammatory and odontogenic cysts), bone-related lesions (ossifying fibroma, fibrous dysplasia and Paget’s disease), traumatic lesions (bony fracture), iatrogenic lesions (lesions associated with surgical procedures), inflammatory lesions (mucosal thickening, retention cysts, opacification, sinus polyps and antrolith), systemic diseases which affect the sinus region, and the silent sinus syndrome disease [11-13]. Data were gathered and divergences between the examiners were solved by reaching a consensus.

**Figure 1:**
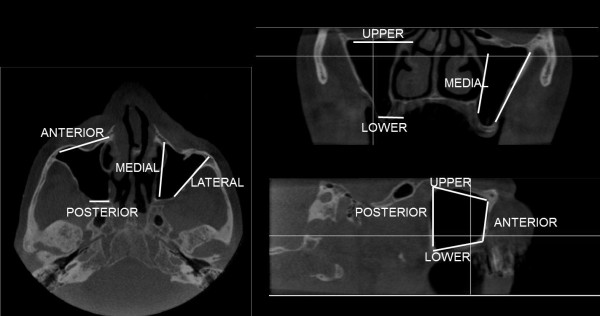
The locations of the abnormalities were recorded using orthogonal view of maxillary sinus.

The location of the diagnosed abnormalities were also recorded as affecting either the anterior, posterior, upper, lower, lateral and/or medial walls of the sinus (Figure 1) and they were classified on based on the method of Nishimura & Iizuka (2002) [14]. When the stored file did not allow for an acceptable view of the entire sinus volume, then that case was excluded from the sample.

The presence of periapical lesions in the upper posterior teeth was recorded and proximity of the lesions to the lower sinus wall was classified using the proposal of OBERLI et al. (2007): class I (near to the sinus floor), class II (in contact with the sinus floor) and class III (overlapping the sinus floor) [15]. The presence of a periapical lesion was recorded when the lamina dura was imperceptible or had an irregular appearance, and when there was a radiolucency indicating bone destruction around the root apex. In cases of multiple periapical lesions near the sinuses, only the most closely related to the sinus was recorded.

The data were analysed using descriptive statistics and the chi-square tests. The inter-rater agreement was calculated using Kappa statistics. SPSS 17.0 software (SPSS Inc., Chicago, IL) was used for the data analysis.

## Results

The CBCT exams of 1113 patients were included in the sample, 678 (60.9%) of which were female and 435 (39.1%) male. Their ages ranged from 12 to 85 years (mean = 49.0; SD = 15.0). The exams had been undertaken for diagnostic purposes, such as implant planning (69.2%), endodontic exam (16%), surgical planning (8.3%), oral disease diagnosis (3.5%), TMJ dysfunction (1.0%), orthodontic diagnosis (0.9%), and traumatology (0.7%).

The first radiologist detected sinus abnormalities in 688 exams (61.8%), while the second detected them in 745 (66.9%). Inter-rater agreement was 92.2% (31.7% without any abnormality and 60.5% with an abnormality) and the kappa coefficient was 0.83, which indicated an excellent degree of agreement in the identification of cases with and without abnormality. Agreement for the identification of abnormal sinuses (right and left sides) was 87.5%, and the kappa coefficient was 0.75. There was a significant difference between the genders, showing a greater occurrence of sinus abnormalities in males: 71.5% versus 55.6% for examiner 1 and 73.8% versus 62.5% for examiner 2 (p < 0.001). No difference in the occurrence of abnormalities was observed with regards to the tercile of age groups (p > 0.05).

When both examiners concluded that there was no abnormality, a third radiologist was requested to confirm the true negative diagnosis at this screening stage. Such cases amounted to 353 (31.7%) and were excluded from the sample. The remaining cases were included because either both examiners (n = 673; 60.5%) or at least one (n = 87; 7.8%) detected some sinus abnormality.

The study sample after screening comprised 760 (68.3%) images with a suspicion of abnormality. At the next stage, 57 images were excluded due to metallic artifacts which result in image noise, thereby impairing visualization and upsetting the classification of the whole image. Thus the final sample size was made up of 703 images (1406 sinuses).

A comprehensive evaluation of the images with sinus abnormalities was performed by the two examiners. Of the 20 abnormalities types, the percentage agreement between the examiners was 53.6% (n = 377) for bilateral (right and left sides), 34.0% (n = 239) for unilateral, and there was complete disagreement in 12.4% (n = 87) of cases. After consensus, the frequency distribution of abnormalities was detailed in Table 1.

**Table 1:** Frequency distribution of sinusal abnormalities in images of 703 patients and 1406 sinuses (n = 1268 lesions)

**Type of abnormalities**	**Right side**	**Left side**	**Total**
Inflammatory			
Mucosal thickening	422 (33.2%)	416 (32.8%)	838 (66.0%)
Retention cysts	49 (3.8%)	81 (6.3%)	130 (10.1%)
Opacification	54 (4.2%)	46 (3.6%)	100 (7.8%)
Sinus Polyps	49 (3.8%)	24 (1.8%)	73 (5.6%)
Antrolith	24 (1.8%)	19 (1.4%)	43 (3.2%)
Iatrogenic			
Oroantral communication	18 (1.4%)	11 (0.8%)	29 (2.2%)
Traumatic			
Fracture	10 (0.7%)	10 (0.7%)	20 (1.4%)
Neoplasia			
Malignant tumours	7 (0.5%)	7 (0.5%)	14 (1.0%)
Odontogenic lesions			
Inflammatory cysts	2 (0.2%)	3 (0.2%)	5 (0.4%)
Odontogenic cysts	2 (0.2%)	2 (0.2%)	4 (0.3%)
Benign odontogenic tumours	3 (0.2%)	1 (0.1%)	4 (0.3%)
Congenital			
Hypoplasia	-	3 (0.2%)	3 (0.2%)
Bone-related lesions			
Fibrous dysplasia	2 (0.2%)	1 (0.1%)	3 (0.2%)
Ossifying fibroma	1 (0.1%)	1 (0.1%)	2 (0.2%)

**Figure 2:**
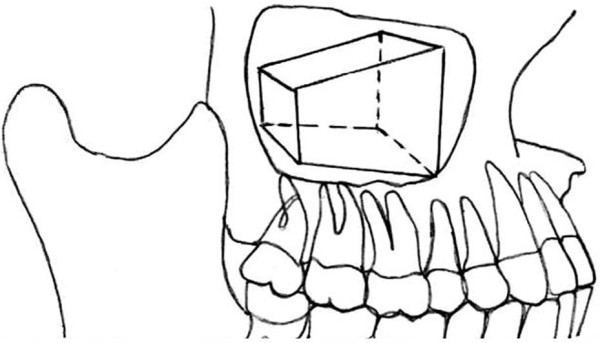
Schematic superposition of the geometric volume of the maxillary sinus and definition of sinus walls.

The location and extension of the abnormalities are schematically illustrated in Figure 2 and Table 2. The frequency distribution from highest to lowest were the inferior (46.2%), anterior (29%), medial (25.7%), lateral (21.5%), posterior (16.6%) and superior walls (5.9%).

**Table 2:** Distribution of wall location of abnormalities within the geometric volume of the sinus (n = 2118)

	Wall locations*											
**Sinuses**												
	(M)	(I)	(L)	(AI)	(API)	(APS)	(PI)	(S)	(AS)	(P)	(A)	(PS)
Right	277	226	229	129	118	56	8	7	5	0	0	0
Left	269	239	226	146	102	50	10	5	3	8	5	0
Total	546	465	455	275	220	106	18	12	8	8	5	0
%	25.8	22.0	21.5	13.0	10.3	5.0	0.8	0.6	0.4	0.4	0.2	0

* Location of the abnormalities within the sinus: *M* – medial wall, *L* – lateral wall, *I* – inferior wall, *S*- superior wall, *A* – anterior wall, *P* – posterior wall, *AI* – anterior and inferior wall, *AS* – anterior and superior wall, *PS* – posterior and superior wall, *PI* – posterior and inferior wall, *APS* – anterior, posterior and superior wall, *API* – anterior, posterior and inferior wall.

Table 3 shows the cross tabulation between the classification of periapical lesions and three types of abnormality (mucosal thickening, opacification and retention cyst). The results showed that there was no difference in the distance of periapical lesion classification according to presence and type of inflammatory abnormality (p = 0.124).

**Table 3:** Frequency of periapical lesion classification of proximity of the sinus inferior wall and type of inflammatory abnormalities (percentage in parenthesis)

**Abnormalities**	**Classification**			
	**Class I**	**Class II**	**Class III**	**Total**
None	10 (19.2)	21 (40.4)	21 (40.4)	52 (100)
Mucosal thickening	26 (19.3)	48 (35.6)	61 (45.2)	135 (100)
Opacification	-	7 (87.5)	1 (12.5)	8 (100)
Retention cyst	3 (15)	6 (30.0)	11 (55.0)	20 (100)
Total	39	82	94	215

p=0.124 (Chi-square test).

## Discussion

MS abnormalities were highly prevalent in this sample of asymptomatic patients. These results emphasized the importance of exploring the entire volume of the CBCT image, including the MS and related areas, and how important it is to consider the whole clinical context when interpreting abnormalities.

It has been widely recognized that the introduction of the CBCT exam was one of the greatest advances in recent years in terms of diagnostic imaging in dentistry. However, an interpretation of CBCT images requires familiarity with the anatomy of the area under investigation, an understanding of the spatial relationships of the image volume, a sound knowledge of the possible diseases, anatomical variations and abnormalities which affect the maxillofacial area and, finally, competence when formulating a differential diagnosis [1,2,16]. These requirements are frequently overlooked and technical difficulties such as the lack of soft tissue contrast often lead to an inability to diagnose soft tissue abnormalities, thereby, increasing the vulnerability of oral-maxillofacial radiologists [2].

A CBCT examination of the maxilla anatomy is commonly requested to evaluate the need of a surgical sinus lift for implant placement in the posterior maxilla. Incidental findings such as mucosal thickening can be associated with a sinus outflow obstruction which can impact on the clinician’s treatment decisions [6]. This abnormality is frequently investigated in exams for implant treatment planning, but other abnormalities are often overlooked.

Several studies have reported a great variability in the prevalence of incidental findings in the maxillary sinuses of asymptomatic subjects when multiplanar images are used. CT scanning studies found abnormalities in approximately 30% of cases [3,4] and CBCT studies reported a prevalence ranging from 24.6% to 56.3% [7-9]. In our study, we detected incidental abnormalities in 68.3% of the cases. Discrepancies in abnormality rates may be due to several factors, such as dissimilarities in the sampling criteria, variations in image interpretation and diagnostic criteria and influence of the climate among differences geographical areas [3,5,17-19].

In our study we found higher prevalence of abnormalities than Ritter et al. (2011) [9], probably due to the fact that we investigated a greater number of possible causes of alterations in maxillary sinus, such as odontogenic lesions, benign and malignant lesions, acquired and congenital lesions, bone related, traumatic and iatrogenic lesions.

Sinus abnormalities were more frequently found in males (p < 0.001). Similar results were reported by Vallo et al. [20], who suggested that the latter are more vulnerable to the occurrence of mucosal thickening due to irritation of the sinus mucosa because of the greater prevalence of pathologic dental findings in males. We detected no influence of age on the occurrence of sinus abnormalities. While our sample covered a wide range of ages, it did not include patients under 12-years old because the formation of their MS is still incomplete and certain abnormalities such as mucosal thickening and opacification are common findings in early childhood and are not indicative of sinus disease [3].

Mucosal thickening was the most frequently observed abnormality (66.0%). It is generally associated with some kind of irritation, such as odontogenic pathology or allergic phenomena [13]. Nonvital posterior maxillary teeth, periodontal abscesses, retained roots, embedded or impacted teeth, extensively carious teeth and oro-antral fistulae could be etiological factors in pathologies of odontogenic origin [20].

Although odontogenic irritation may be potentially influenced by the proximity between roots with periapical lesions and the floor of the sinus [12,20,21], we did not find any significant association. In addition, there is no consensus in the literature on the amount of mucosal thickening considered abnormal. Previous studies have suggested measurements ranging from 2 to 6 millimeters [6,10,18,20,22,23]. As we considered 3 mm as the reference measure, this could also have influenced the high occurrence of mucosal thickening in our study [23].

The lower sinus wall was the most affected location within the sinus, which would suggest a possible odontogenic involvement [22]. However, considering that the CBCT increases the accuracy of detecting periapical lesions [24,25], these results should be interpreted with caution, since incipient and chronic periapical lesions detected by a CBCT exam might present low potential for evocating sinusal inflammatory signs and symptoms. The low prevalence of abnormalities in the upper sinus wall may also have been influenced by the limited visualization of this region due to the small FOV (6 cm) commonly used for this examination.

Mucous retention cysts were the second most frequently found inflammatory abnormality (10.1%). This result is similar to other studies [19,26,27] of general dental patients, when plain panoramic radiography was used and a prevalence ranging from 1.4% to 9.6% was found. Other study [28] using sectional exams obtained by CT and MRI reported a prevalence of 12.4%.

Opacification was observed in 7.8% of the exams, which would suggest an occurrence of sinusitis. However, sometimes opacification can also be found in abnormalities other than sinusitis, such as mechanical trauma, barotraumas and hemorrhage [13]. In addition, a final diagnosis of sinusitis may also be considered when clinical signs and symptoms are present and such factors were not evaluated in this study [29,30].

## Conclusion

The high occurrence of abnormalities in asymptomatic MS emphasizes how important it is for the dentomaxillofacial radiologist to undertake a comprehensive interpretation of the whole volume acquired in CBCT images, including the entire MS when analyzing the imaging exams of routine patients. Incidental findings may be considered in the individual clinical context of signs and symptoms, reducing the risk of overestimation of the real impact of radiographic findings.

## Competing interests

The authors declare that they have no competing interests.

## Authors’ contribution

All of the listed authors contributed to the conduct of the study. ICCR and TOS analyzed and interpreted the CBCT images and drafted the manuscript. CRL and EFM contributed to the overall conceptualization and design of the study. All authors contributed to the writing of the final version and have read and approved the final manuscript.

## Pre-publication history

The pre-publication history for this paper can be accessed here:

http://www.biomedcentral.com/1472-6831/12/30/prepub

## References

[B1] Carter L Farman AG Geist J Scarfe WC Angelopoulos C Nair MK American Academy of Oral and Maxillofacial Radiology Executive Opinion Statement on Performing and Interpreting Diagnostic Cone Beam Computed Tomography Oral Surg Oral Med Oral Pathol Oral Radiol Endod 2008 106 561 562 10.1016/j.tripleo.2008.07.007 18928899

[B2] Campbell PD Zinreich SJR Aygun N Imaging of the paranasal sinuses and In-Office CT Otolaryngol Clin North Am 2009 42 753 764 10.1016/j.otc.2009.08.015 19909856

[B3] Diament MJ Senac MOJR Gilsanz V Baker S Gillespie T Larsson S Prevalence of incidental paranasal sinuses opacification in pediatric patients: a CT study J Comput Assist Tomogr 1987 11 426 431 10.1097/00004728-198705000-00011 3571583

[B4] Havas TE Motbey JA Gullane PJ Prevalence of incidental abnormalities on computed tomographic scans of the paransal sinuses Arch Otolaryngol Head Neck Surg 1988 114 856 859 10.1001/archotol.1988.01860200040012 3390327

[B5] Lesserson JA Kieserman SP Fin DG The radiographic incidence of chronic sinus disease in the pediatric population Laryngoscope 1994 104 159 166 8302118 10.1288/00005537-199402000-00007

[B6] Carmeli G Artzi Z Kozlovsky A Segev Y Landsberg R Antral computerized tomography pre-operative evaluation: relationship between mucosal thickening and maxillary sinus function Clin Oral Impl Res 2011 22 78 82 10.1111/j.1600-0501.2010.01986.x 20946209

[B7] Cha J-Y Mah J Sinclair P Incidental findings in the maxillofacial area with 3-dimensional cone-beam imaging Am J Orthod Dentofacial Orthop 2007 132 7 14 10.1016/j.ajodo.2005.08.041 17628245

[B8] Pazera P Bornstein MM Pazera A Sendi P Katsaros C Incidental maxillary sinus findings in orthodontic patients: a radiographic analysis using cone-beam computed tomography (CBCT) Orthod Craniofac Res 2011 14 17 24 10.1111/j.1601-6343.2010.01502.x 21205165

[B9] Ritter L Lutz J Neugebauer J Scheer M Dreiseidler T Zinser MJ Rothamel D Mischkowski RA **Prevalence of pathologic findings in the maxillary sinus in cone-beam computerized tomography** Oral Surg Oral Med Oral Pathol Oral Radiol Endod 2011 111 634 340 10.1016/j.tripleo.2010.12.007 21444226

[B10] Mehra P Murad H Maxillary sinus disease of odontogenic origin Otolaryngol Clin North Am 2004 37 347 364 10.1016/S0030-6665(03)00171-3 15064067

[B11] Lawson W Patel ZM Lin FY The development and pathologic processes that influence maxillary sinus pneumatization Anat Rec 2008 291 1154 1163 10.1002/ar.20774 18951496

[B12] Mehra P Jeong D Maxillary sinusitis of odontogenic origin Curr Allergy Asthma Rep 2009 9 238 243 10.1007/s11882-009-0035-0 19348725

[B13] Madani G Beale TJ Sinonasal inflammatory disease Semin Ultrasound CT MRI 2009 30 17 24 10.1053/j.sult.2008.10.012 19388235

[B14] Nishimura T Iizuka T Evaluation of the pathophysiology of odontogenic maxillary sinusitis using bone scintigraphy Int J Oral Maxillofac Surg 2002 31 389 396 10.1054/ijom.2001.0198 12361072

[B15] Oberli K Bornstein MM Von Arx T Periapical surgery and the maxillary sinus: radiographic parameters for clinical outcome Oral Surg Oral Med Oral Pathol Oral Radiol Endod 2007 103 848 853 10.1016/j.tripleo.2006.09.017 17197213

[B16] Scarfe WC Farman AG Sukovic P Clinical applications of cone-beam computed tomography in dental practice J Can Dent Assoc 2006 72 75 79 16480609

[B17] Bolger WE Butzin CA Parsons DS Paranasal sinus bony anatomic variations and mucosal abnormalities: CT analysis for endoscopic sinus surgery Laryngoscope 1991 101 56 64 1984551 10.1288/00005537-199101000-00010

[B18] Lim W-K Ram B Fasulakis S Kane KJ Incidental magnetic resonance image sinus abnormalities in asymptomatic Australian children J Laryngol Otol 2003 117 969 972 14738607 10.1258/002221503322683858

[B19] Rodrigues CD Freire GF Silva LB Fonseca da Silveira MM Estrela C Prevalence and risk factors of mucous retention cysts in a Brazilian population Dentomaxillofac Radiol 2009 38 480 483 10.1259/dmfr/48774803 19767520

[B20] Vallo J Taipale LS Huumonen S Soikkonen K Norblad A Prevalence of mucosal abnormalities of the maxillary sinus and their relationship to dental disease in panoramic radiography: results from the Health 2000 Hearth Examination Survery Oral Surg Oral Med Oral Pahtol Oral Radiol Endod 2010 109 e80 e87 10.1016/j.tripleo.2009.10.031 20219592

[B21] Hauman CHJ Chandler NP Tong DC Endodontic implications of the maxillary sinus: a review Int Endod J 2002 35 127 141 10.1046/j.0143-2885.2001.00524.x 11843967

[B22] Obayashi N Ariji Y Goto M Izumi M Naitoh M Kurita K Spread of odontogenic infection originating in the maxillary teeth: computerized tomographic assessment Oral Surg Oral Med Oral Pathol Oral Radiol Endod 2004 98 223 231 10.1016/j.tripleo.2004.05.014 15316549

[B23] Gordts F Clement PAR Destryker B Desprechins B Kaufman L Prevalence of sinusitis signs on MRI in a non-ENT paediatric population Rhinology 1997 35 154 157 9532633

[B24] Lofthag-Hansen S Huumonen S Grondahl K Grondahl HG Limited cone-beam CT and intraoral radiography for the diagnosis of periapical pathology Oral Surg Oral Med Oral Pathol Oral Radiol Endod 2007 103 114 119 10.1016/j.tripleo.2006.01.001 17178504

[B25] Low KMT Dula K Burgin W von Arx T Comparison of periapical radiography and limited cone-beam tomography in posterior maxillary teeth referred for apical surgery J Endod 2008 34 557 562 10.1016/j.joen.2008.02.022 18436034

[B26] Bósio JA Tanaka O Rovigatti E Gruner SK The incidence of maxillary sinus retention cysts in orthodontic patients World J Orthod 2009 10 e7 e8 19582248

[B27] Rhodus NL The prevalence and clinical significance of maxillary sinus mucous retention cysts in a general clinic population Ear Nose Throat J 1990 69 82 87 2311544

[B28] Bhattacharyya N Do maxillary sinus retention cysts reflect obstructive sinus phenomena? Arch Otolaryngol Head Neck Surg 2000 126 1369 1371 11074835 10.1001/archotol.126.11.1369

[B29] Hansen JG Lund E The association between paranasal computerized tomography scans and symptoms and signs in a general practice population with acute maxillary sinusitis APMIS 2010 119 44 48 21143525 10.1111/j.1600-0463.2010.02690.x

[B30] Mudgil SP Wise SW Hopper KD Kasales CJ Mauger D Fornadley JA Correlation between presumed sinusitis-induced pain and paranasal sinus computed tomographic findings Ann Allergy Asthma Immunol 2002 88 223 226 10.1016/S1081-1206(10)62000-5 11868929

